# High Output Heart Failure in Multiple Myeloma: Pathogenetic Considerations

**DOI:** 10.3390/cancers14030610

**Published:** 2022-01-26

**Authors:** Melania Carlisi, Salvatrice Mancuso, Rosalia Lo Presti, Sergio Siragusa, Gregorio Caimi

**Affiliations:** 1 Department of Health Promotion, Mother and Child Care, Internal Medicine and Medical Specialties, University of Palermo, 90127 Palermo, Italy; salvatrice.mancuso@unipa.it (S.M.); sergio.siragusa@unipa.it (S.S.); gregorio.caimi@unipa.it (G.C.); 2 Department of Psychology, Educational Science and Human Movement, University of Palermo, 90127 Palermo, Italy; rosalia.lopresti@unipa.it

**Keywords:** high output heart failure, multiple myeloma, artero-venous fistulae, angiogenesis, glutamminolysis, hyperammonemia, plasma viscosity

## Abstract

**Simple Summary:**

Multiple myeloma is a plasma cell disorder that accounts for around 10% of all haematological malignancies. This neoplasia is often associated with a significant prevalence of cardiovascular complications resulting from several factors, unrelated and/or related to the disease. Among cardiovascular complications, the high output heart failure is of great importance as it is related to a worse prognosis for patients. It is important to point out that, despite the availability of more and more numerous and effective drugs, myeloma remains an incurable disease, with frequent relapses and several treatment lines, with the need, therefore, for a careful evaluation of patients, especially from a cardiological point of view. For this reason, we are proposing a comprehensive overview of different pathogenetic mechanisms responsible for high output heart failure in multiple myeloma, including artero-venous shunts, enhanced angiogenesis, glutamminolysis, hyperammonemia and hemorheological alterations, with the belief that a multidisciplinary approach, in clinical evaluation is critical for the optimal management of the patient.

**Abstract:**

The high output heart failure is a clinical condition in which the systemic congestion is associated to a high output state, and it can be observed in a non-negligible percentage of hematological diseases, particularly in multiple myeloma, a condition in which the risk of adverse cardiovascular events may increase, with a worse prognosis for patients. For this reason, though an accurate literature search, we provided in this review a complete overview of different pathogenetic mechanisms responsible for high output heart failure in multiple myeloma. Indeed, this clinical finding is present in the 8% of multiple myeloma patients, and it may be caused by artero-venous shunts, enhanced angiogenesis, glutamminolysis, hyperammonemia and hemorheological alterations with increase in plasma viscosity. The high output heart failure in multiple myeloma is associated with significant morbidity and mortality, emphasizing the need for a multidisciplinary approach.

## 1. Introduction

**Figure 1:**
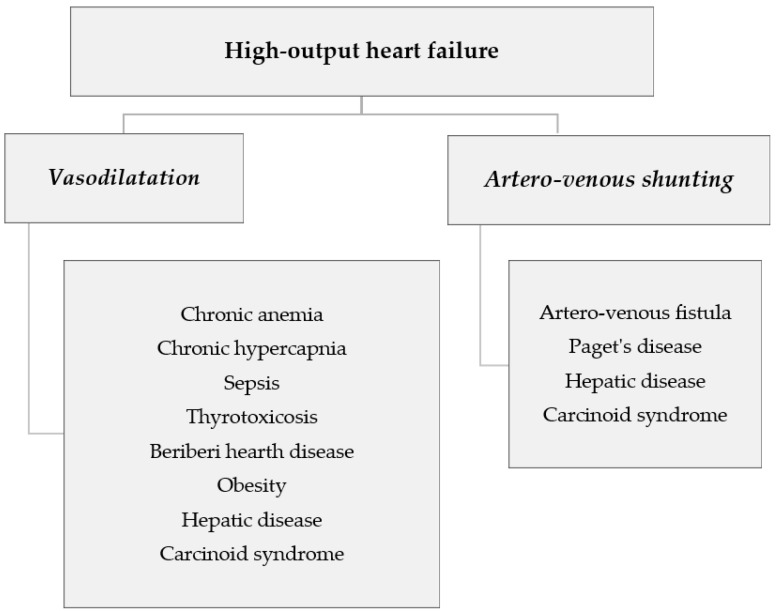
Pathogenetic mechanisms and common causes of high-output heart failure.

High output heart failure (HOHF) is a clinical condition in which the systemic congestion is associated with a high output state, and it is characterized, at rest, by a cardiac output greater than 8 L/min or a cardiac index > 3.9 L/min/m^2^ [1,2,3]. The cardiac index is a cardiovascular hemodynamic parameter, and it is obtained by dividing the cardiac output by the body surface area with a normal range of 2.2 to 3.5 L/min/m^2^. Congestive heart failure is a complex syndrome with several symptoms and signs, including dyspnea, increased fatigability, tachypnea, tachycardia, pulmonary rales, and peripheral edema. Usually, this syndrome is associated with low cardiac output, but it may also occur in several so-called high output states, with a normal, or greater than normal, cardiac output. Usually, the most common causes of HOHF, through pathogenetic mechanisms of vasodilatation and/or artero-venous shunting, include severe anemia, arteriovenous fistulas (acquired and/or congenital), Paget’s disease with bone involvement exceeding 15%, chronic hypercapnia, hyperthyroidism and thyrotoxicosis, sepsis, beriberi, obesity during pregnancy, chronic liver disease and carcinoid syndrome (Figure 1). Furthermore, HOHF can be found [4] in a non-negligible percentage (8%) of patients with hematological disorders, particularly in multiple myeloma (MM).

MM is the second most common hematologic malignancy, and it is included in the spectrum of plasma cell dyscrasias. It is preceded in most cases by a precursor disease, with gammopathy of unknown significance (MGUS) being the most frequent. Primary plasma cell leukemia is a rare and more aggressive form of MM. MM is a disease more frequently observed in the older population, and it is associated with significant morbidity due to its end-organ destruction, including possible cardiovascular complications.

HOHF has been described in patients with MM repeatedly, but it is often underestimated in this disease. It occurs because of reduced vascular resistance associated with a compensatory increase in cardiac output. Generally, symptoms include dyspnea, at rest and/or under exertion, intolerance to physical exercise, asthenia, and hydro-saline retention, while the objective data includes tachycardia, tachypnea, increased jugular venous pressure, pulmonary rales, pleural effusion, and peripheral oedema.

As is known, the diagnosis of HOHF is carried out with the use of a teleradiogram, an accurate echocardiographic examination and a blood gas measurement, even if the gold standard is certainly invasive hemodynamic investigation. The diagnosis must be particularly accurate because patients with HOHF show a marked increase in mortality [4], with a prognosis that seems to vary according to the different responsible causes. The treatment for each clinical disorder responsible for HOHF turns out to be different even if the hydro-saline retention therapy can only include the use of diuretics under constant and careful monitoring of the electrolytes.

In the case of MM, it should be remembered that many patients may present cardiovascular risk factors and/or comorbidities at diagnosis and, in addition, the disease itself can have negative effects, direct or indirect, on cardiac function. It should also be considered that MM patients are usually exposed to different types of treatments, usually with the association of several drugs, and each of them can increase the risk of adverse cardiovascular events. Furthermore, in patients with MM the incidence of venous thromboembolism varies from 3% to 10%, the possibility of congestive heart failure is around an average of 8% and those who have received steroid treatment and at least three different anti-MM therapies have arterial hypertension in 36% of cases. Another noticeable complication during the treatment of these patients appears to be the presence of elongated QT on the ECG evaluation [5]. For this reason, it is very important to perform a careful assessment of the cardiovascular risk in these patients, both before and during the treatment, exploiting a multidisciplinary approach with a cardio-oncological support [6].

**Figure 2:**
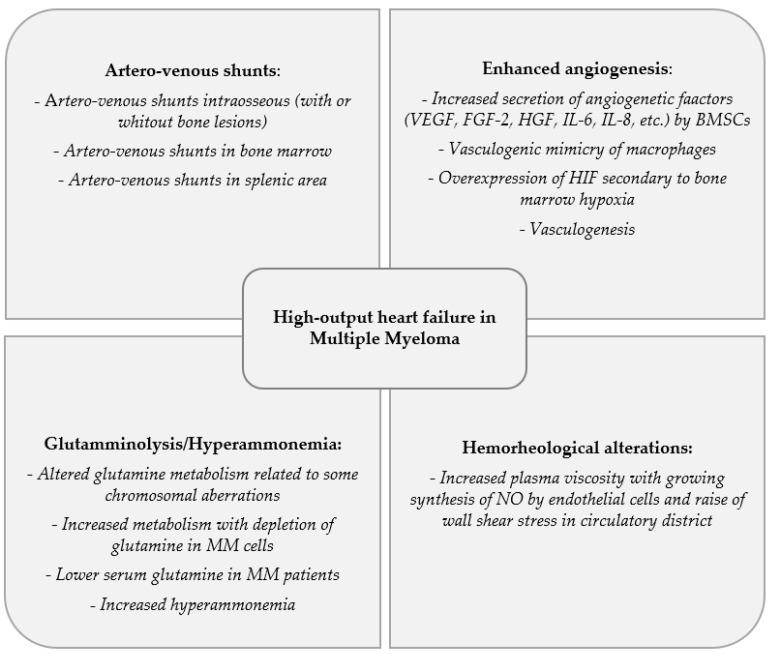
Factors linked to the high-output heart failure (HOHF) in Multiple Myeloma.

In this manuscript, our aim is to provide a complete nosographic framework about the conditions that can cause an HOHF in MM patients. Some papers in the literature have tried to describe the possible etiologies of HOHF, but, in the specific case of MM, this review would be the first paper in which a complete examination of the possible pathways of HOHF is provided. Among pathogenetic mechanisms considered responsible for the development of HOHF in MM patients, we can find artero-venous shunts, enhanced angiogenesis, glutamminolysis and hyperammonemia and hemorheological alterations (Figure 2).

## 2. Methods

We consulted MEDLINE (PubMed) electronic databases for English language papers using a list of specific search terms, such as “high output cardiac failure” and “cardiac failure and multiple myeloma”. Papers were also screened in the reference list of relevant reviews/articles. Original articles, reviews, case reports, case series and letters to the editor from all years of publication have been reviewed.

## 3. Artero-Venous Shunts

From the moment in which the first reports of HOHF in patients with MM occur [7,8] it was considered that the possible pathogenetic moment of this altered hemodynamic structure must largely refer to the presence of artero-venous shunt within the bone lesions that accompany the course of the disease often. Therefore, this hypothesis led some authors to believe that the presence of a condition of HOHF was almost exclusive to MM cases with an extensive and marked bone involvement. In this regard, in a clinical research [9] including 34 patients with MM, 8 presented a high cardiac output evaluated with the use of a two-dimensional echocardiography integrated using pulsed doppler, with a bone involvement resulting in 100% of patients; in the group without HOHF (26 patients), the bone involvement was only 35%. Other authors, after evaluating the cardiac output with a pulsed doppler echocardiograph in a group of 28 MM patients, had also theorized that in patients with MM, the only risk factor for the onset of a syndrome of high cardiac output was the presence of osteolytic lesions [10].

Other researchers [11] had demonstrated in 11 patients with MM—with the scintigraphy technique carried out with the use of Tc-labeled albumin macroaggregates—an artero-venous shunt equal to almost 19%, and they had described a significant and positive correlation between the values of the shunt and the cardiac index.

However, the presence of artero-venous shunts in patients with MM with a central venous saturation greater than 75%—cannot be referred only to the osteolytic areas; almost all are present in the flat bones that are not accompanied by any periosteal reaction.

Moreover, it should be remembered that the marked angiogenic activity, particularly in the intramedullary area, is responsible for innumerable artero-venous fistulas within the bone marrow, which can facilitate the hemodynamic condition underlying the HOHF observable in MM patients. Furthermore, other authors [12] have considered that the site of the artero-venous shunt may also be in the splenic area. In this regard, it has been hypothesized that the increase in splenic flow secondary to splenomegaly could contribute to the hemodynamic condition of high cardiac output. Indeed, the splenic blood flow can represent almost 55% of the entire cardiac flow [13]. However, it must be underlined that splenomegaly is very rare in MM patients, and the few described cases in the literature are those of patients with a simultaneous presence of acquired hemophagocytic lymphohistiocytosis, myeloproliferative neoplasms and AL amyloidosis [14,15,16].

Finally, cases of patients with plasma cell dyscrasia (specifically plasma cell leukemia) with an HOHF condition but without documented osteolytic lesions are also documented in the literature [17,18]. For these reasons, the role of intraosseous artero-venous shunts as a main contributing cause of high cardiac output has been resized.

## 4. Enhanced Angiogenesis

Another possible pathogenetic mechanism of HOHF can be found in the altered and marked angiogenetic activity that characterizes MM, a very interesting topic in recent years analyzed by various research groups [19,20,21,22,23,24,25,26,27].

Angiogenesis represents the production of new vessels from a pre-existing vasculature that happens in both physiological and pathological conditions. This event begins with a vasodilation and with an increased permeability of existing vessels. The alteration of the surrounding matrix allows the activated and proliferating endothelial cells to migrate and develop lumens. Later, these cells differentiate and mature into an intricate complex of vessels, sustained by peri-endothelial cells and matrix.

In physiological conditions, angiogenesis depends on the balance of positive and negative angiogenic modulators. In neoplastic conditions, the angiogenesis is due to a switch of this balance, and it mainly depends on the production of growth factors able to stimulate the growth of the host’s blood vessels [28]. Several papers have demonstrated that the degree of angiogenesis and/or the levels of angiogenic factors are related to disease stage and its prognosis, suggesting that angiogenesis is a key factor in control of tumor progression [29].

In hematological diseases, the bone marrow angiogenesis plays an important role in the pathogenesis and progression of the malignancies. It is well known that tumor microenvironment promotes angiogenesis, proliferation, invasion, and metastasis and mediates mechanisms of therapeutic resistance [30].

In MM, the angiogenesis is an important feature, especially in the progression from MGUS to MM. It is induced by angiogenic factors released by cells composing the microenvironment with a specific prognostic value [31].

It is important to underline that normal plasma cells express a lot of pro-angiogenic factors, able to induce in vitro angiogenesis. This angiogenic stimulus is further increased in MM, due to the aberrant expression of pro-angiogenic and downregulation of anti-angiogenic genes by plasma cells. Rajkumar et al. [32] have demonstrated, in a group of 400 patients, a progressive increase in bone marrow microvascular density in several plasma cell disorders, including primary amyloidosis, MGUS, smoldering and active MM. Other papers have described a correlation between bone marrow (BM) microvascular density and progression-free survival and overall survival in MM patients [33]. Moreover, BM microvascular density decreases significantly in patients achieving a remission, but not in patients who had no response to therapy [34].

Within the BM microenvironment, stromal cells (BMSCs), hematopoietic stem cells (HSCs), fibroblasts, osteoblasts/osteoclasts, adipocytes, endothelial precursor cells (EPCs), T lymphocytes, macrophages, and mast cells increase the concentration of angiogenic factors by direct secretion or after stimulation by myeloma cells or endothelial cells, through paracrine interactions. BMSCs, osteoclasts, osteoblasts and endothelial cells secrete several molecules, including vascular endothelial growth factor (VEGF), fibroblast growth factor-2 (FGF-2), tumor necrosis factor alpha (TNF-alpha), hepatocyte growth factor (HGF), interleukin-6 and -8, osteopontin (OPN), angiopoietin-1 (Ang-1), B-cell activating factor, stromal cell-derived factor 1 (SDF-1), and various Notch-family members [35].

MM cells secrete VEGF and FGF-2 that induce the cells of the tumor microenvironment to secrete their own VEGF, FGF-2, and HGF, able to recruit and activate the MM-associated macrophages. In active MM, bone marrow macrophages contributed to the angiogenesis through a vasculogenic mimicry. When exposed in vitro to VEGF and FGF-2, tumor macrophages differentiated into cells like MM endothelial cells, able to generate in vitro capillary-like networks.

Among factors inducing the angiogenesis phenomenon in MM, an important role is played by the hypoxia-inducible factor (HIF). As it is known, HIF is a heterodimer comprising HiF-1 alpha and HiF-1 beta subunits, both of which are basic helix-loop-helix transcription factors. HiF-1 beta is a nuclear protein constitutively expressed independent of O_2_ tension, while HiF-1 alpha is a cytoplasmatic protein responsive to O_2_ tension. Generally, the BM is hypoxic and the pO2 measured in bone marrow aspirates is of 54.9 mmHg, with a mean O2 saturation of 87.5% [36]. This hypoxic condition found in BM microenvironment, also observed in MM patients [37,38,39], leads to an overexpression of HIF.

Besides the angiogenesis, the new vessels in the bone marrow of MM patients are due to the vasculogenesis; this process is a “de novo” synthesis of blood small vessels from the endothelial progenitor cells (EPC), enrolled from the bone marrow. The EPCs are incorporated into nascent vessels at the angiogenic locations, and they proliferate and differentiate into endothelial cells [40]. Circulating EPCs and endothelial cells are observed generally in MM patients, and their concentration seems to correlate with disease activity.

The importance of the angiogenesis process in MM is such that, also from a therapeutic point of view, it has been tried to act on this important pathway, using drugs with anti-angiogenetic activity. Among these drugs, we must allude to thalidomide, lenalidomide, pomalidomide and bortezomib. The anti-angiogenetic activity of thalidomide is due to the inhibition of secretion of VEGF and IL-6. Moreover, thalidomide enhances T cell- and NK cell-mediated immunological responses and induces caspase-8 mediated apoptosis [41]. Subsequently to Thalidomide, a series of immunomodulatory drugs have been developed, with an important anti-angiogenetic potential, including Lenalidomide e Pomalidomide. Lenalidomide inhibits the VEGF-induced PI3K-Akt pathway signaling and the HIF-1alfa expression [42], exerts an anti-TNF-alpha activity, modulates the immune response stimulating T cells and NK cells activities, induces apoptosis of tumor cells, and decreases the binding of MM cells to stromal cells [43]. Pomalidomide is active against MM cell lines and inhibits angiogenesis by targeting VEGF and HIF-1 alpha. Bortezomib induces endothelial cell apoptosis, inhibits VEGF, IL-6, Ang-1 and Ang-2 and IGF-1 secretion in stromal cells and endothelial cells in MM patients. Finally, in the field of the support therapy, the administration of bisphosphonates, inhibitors of osteoclasts activity, also brings into play an antiangiogenic activity [27].

## 5. Glutamminolysis and Hyperammonemia

Generally, MM cells are characterized by an increased metabolism of glucose, glutamine, fatty acids, and nucleotides, principally mediated by the activation of specific oncogenes [44]. In addition, in vitro, the MM cells growth is strictly bounded by the depletion of glutamine, suggesting that glutamminolysis has a key role in the complex metabolism of these cells [45,46]. At the same time, it has been demonstrated that MM patients show significantly lower serum glutamine if compared to healthy controls [47], and recent data point out that the altered glutamine metabolism activated by glutamminolysis [48,49,50] seems to be a metabolic consequence of recurrent chromosomal aberrations observed in MM.

Glutamine, through the action of glutaminase, produces ammonia and therefore ammonaemia; this biochemical cycle tends to explain the real reason why, in recent decades, in patients with MM, but also in patients with plasma cell leukemia and in myeloma cell cultures, a condition of hyperammonemia has been described. This condition may be responsible for a persistent or transient hyperammonemic encephalopathy unresponsive to the treatment used for the encephalopathy of patients with severe hepatic insufficiency, and largely reversible with the pharmacological treatment of MM [51,52,53,54,55,56,57,58,59,60,61,62,63,64,65,66,67,68,69].

The disturbances of consciousness observed in patients with MM are almost always attributable to hypercalcemia or hyperviscosity syndrome, but if both are excluded, in relation to the above, the determination of ammonia levels is decisive.

Although plasma levels of ammonia are quite low, its turnover is very high, while the percentage of its ionized (NH4^+^) and non-ionized (NH3^+^) forms is solely a function of pH levels. Ammonia in humans can be removed through three suitable ways, to eliminate it from cell fluids and incorporate it into compounds with lower basicity and toxicity [70]. The first way, catalyzed by glutamate dehydrogenase, transforms ammonia into glutamate [71]; the second pathway, catalyzed by glutamine-synthetase, occurs in two steps, and synthesizes glutamine starting from glutamate [72]; the third way instead is that of urogenesis. The latter can eliminate about 20 g/die of urea by itself and takes place partly within the mitochondrion and partly in the cytosol. Urea is formed from ammonia, CO_2_ and aspartate through a reaction catalyzed by five specific enzymes involving six amino acids. The final reaction of this cycle is the hydrolytic cleavage of the guanidine group of arginine catalyzed by hepatic arginase. However, it should be emphasized that arginine is also the physiological substrate of endothelial-Nitric Oxide Synthase (eNOS) whose final products are nitric oxide (NO) and citrulline. e-NOS, whose functional activity depends on the presence of high levels of calcium ions, is a particularly complex enzyme which includes five cofactors, such as NADPH, FAD, FMN, heme and tetrahydrobiopterin [73]. In addition to releasing vascular smooth muscle, nitric oxide influences neurotransmission but also the relaxation of skeletal muscle and, particularly, inhibits adhesion, activation, and platelet aggregation.

It is not possible to exclude that, in a percentage of MM patients and in the presence of specific chromosomal aberrations [44], the activation of glutamminolysis of myeloma cells—almost never associated with the increase in peripheral glutamine levels—may be the trigger of an exponential synthesis of ammonia which increases its serum levels with clinical effects on the central nervous system (nonhepatic hyperammonemic encephalopathy). The presence of high levels of ammonia, in the presence of normal liver function, activates its various disposal routes, the main of which involves a marked activity of urogenesis with a circular and accelerated synthesis of arginine that, as reiterated previously, is the natural precursor of e-NOS, and therefore of NO. This possible hypothesis—considering the various reports of patients with MM who present a picture of hyperammonemic encephalopathy or of myeloma patients in which there is a hyperammonemia associated with a clinical-instrumental picture of HOHF with acceptable hemoglobin values—deserves to be considered and appropriately investigated. The role played by arginine as a precursor of eNOS is explicitly confirmed in studies carried out in patients with familial hypercholesterolemia [74], or in patients with type 2 diabetes mellitus [75] in which the inhibition of hepatic arginase leads to a marked improvement in endothelial function.

## 6. Hemorheological Alterations

So far, the alteration of blood viscosity that usually accompanies and characterizes the plasma cell dyscrasias [76,77,78,79,80,81,82] has not been mentioned among the pathogenetic hypotheses considered to explain the hemodynamic condition underlying the HOHF. In addition to the close relationship between hemorheological determinants and microcirculation [83], of particular interest are the links between the hemorheological profile and endothelial function, which have been discussed a lot so far [84,85,86].

Previous studies [87,88], conducted on experimental models, had shown that the increase in plasma viscosity was responsible for the increased synthesis of NO. It is now known that variations in wall shear stress (WSS) related to blood and plasma viscosity actively influence the endothelial activity of eNOS. In this regard, it should be remembered that the determination of the WSS can be carried out both with in vitro and in vivo assessments [89], and it has limitations related to the different sections of the vascular tree in which the analysis is performed; the evaluation of the WSS can also be done on the microcirculation [90].

Some authors [91] have outlined the trigger role of shear stress on the regulation of endothelial function, and they have underlined how flow shear stress plays its role on eNOS activity. Other researchers [92] pointed out that in experimental models of endothelial cell cultures, only a physiological flow, with variable shear stress, was able to significantly increase the activity of the eNOS. At the same time, it was found that a nonlaminar and irregular blood flow was more suitable for promoting the activity of eNOS through endothelial mechano-transduction [93].

From what has been reported so far, NO is synthesized by endothelial cells exposed to mechanical forces and to WSS and intraluminal pressure [94]. According to other researchers [95], mechanical endothelial stimulation activates, in cascade, different and complex biochemical reactions that set in motion different cellular mechanosensors and as many enzymes; only the last step of this cascade is the activation of the eNOS, which catalyzes the oxidation of arginine with the consequent production of NO.

Plasma viscosity also plays an important role in MM patients; in fact, the increase in plasma viscosity causes the shear stress to vary, especially at the level of the microcirculatory district, and this variation reverberates on the endothelial function with all the consequences described so far. It has been said that myeloma cells, through the enhanced activity of glutamminolysis, increase the levels of ammonaemia, and the latter largely uses the ureagenesis pathway with arginine as the final metabolite; in turn, arginine can be transformed in ornithine by hepatic arginase or it be a NO precursor.

This double possibility has not so far been considered in the singular and specific cardiovascular profile that can be associated with MM.

It is in fact possible that the disposal activity of the high levels of ammonia, associated with the increase in plasma viscosity, with the inevitable repercussions on endothelial function, may contribute to a high synthesis of NO, whose immediate effects on the cardiovascular system could alter the hemodynamic structure of patients with MM with HOHF. Therefore, when suspected, HOHF must be ascertained considering that its onset in these patients significantly worsens the prognosis.

## 7. Conclusions

HOHF is a clinical condition characterized by a systemic congestion with a high output state. The most common causes of HOHF include severe anemia, arteriovenous fistulas (acquired and/or congenital), Paget’s disease, chronic hypercapnia, hyperthyroidism with thyrotoxicosis, sepsis, beriberi, obesity during pregnancy, chronic liver disease and carcinoid syndrome.

HOHF can be observed also in different hematological diseases, including MM, a plasma cell dyscrasia characterized by an increased risk of cardiovascular events, determined by both the disease itself and some specific drugs using treatment protocol. Because cardiovascular events represent a significant factor related to poor prognosis, impacting survival of patients and quality of life, it is very important to perform a careful assessment of the cardiovascular risk in MM patients, before and during the treatment. However, at the same time, for a complete clinical evaluation, it is also important to know the possible determinants of cardiological alterations, including HOHF.

For this reason, in this paper we have provided an overview of different pathogenetic mechanisms possibly responsible for HOHF in multiple myeloma. The need to cite specific papers, able to accurately delineate some clinical and pathophysiological characteristics of HOHF in MM (sometimes), has led us to include several quite old papers in the references list. This latter could be a possible limitation of the paper, but in our opinion, it does not seem to diminish the significance of this review.

Up to now, to explain the pathogenesis of HOFH in multiple myeloma, both the presence of artero-venous fistulas (bone, intramedullary or splenic) and the altered angiogenesis have been considered. In relation to the role of enhanced angiogenesis, the specific treatment with anti-angiogenic drugs resulted in a significant improvement in the hemodynamic profile.

In addition, in some plasma cell dyscrasia, complicated or not by HOHF, a condition of hyperammonemia, without liver changes, has been described—one that, indirectly, may be involved in the pathogenesis of this cardiovascular condition.

Finally, the same consideration seems to apply to plasma viscosity which increasing the wall shear stress modifies the functional activity of the eNOS.

The possible involvement of different pathogenic mechanisms in the development of HOHF in MM highlights the complexity of this clinical condition and, therefore, the need for a multidisciplinary approach in the clinical management of patients. Indeed, in future directions, we look forward to obtaining a complete knowledge of patients, with the aim of guaranteeing them, in addition to an optimal treatment strategy, also the best support therapy, with significant impact on quality of life.

## References

[1] Anand I.S., Florea V.G. (2001). High output cardiac failure. Curr. Treat. Options Cardiovasc. Med..

[2] Mehta P.A., Dubrey S.W. (2009). High output heart failure. QJM.

[3] Singh S., Sharma S. (2021). High-Output Cardiac Failure. Current Treatment Options in Cardiovascular Medicine.

[4] Reddy Y.N.V., Melenovsky V., Redfield M.M., Nishimura R.A., Borlaug B.A. (2016). High-Output Heart Failure: A 15-Year Experience. J. Am. Coll. Cardiol..

[5] Plummer C., Driessen C., Szabo Z., Mateos M.-V. (2019). Management of cardiovascular risk in patients with multiple myeloma. Blood Cancer J..

[6] Mancuso S., Carlisi M., Sarocchi M., Napolitano M., Siragusa S. (2018). Cardio-oncology in multiple myeloma: Is it time for a specific focus?. Leuk. Lymphoma.

[7] McBride W., Jackman J.D., Gammon R.S., Willerson J.T. (1988). High-output cardiac failure in patients with multiple myeloma. N. Engl. J. Med..

[8] Kosisnski D.J., Roush K., Fraker T.D., Grubb B.P. (1994). High cardiac output state in patients with multiple myeloma: Case report and review of the literature. Clin. Cardiol..

[9] McBride W., Jackman J.D., Grayburn P.A. (1990). Prevalence and clinical characteristics of a high cardiac output state in patients with multiple myeloma. Am. J. Med..

[10] Lopez L., Portero J.A., Borrego D., Barez A., San Miguel J.F. (1997). High cardiac output in myeloma patients. Its prevalence and clinical characteristics. The Castile-Leon Cooperative Group for the Study of Monoclonal Gammapathies. Med. Clin..

[11] Inanir S., Haznedar R., Atavci S., Unlü M. (1998). Arteriovenous shunting in patients with multiple myeloma and high-output failure. J. Nucl. Med..

[12] Leporrier M. (1989). High-Output Cardiac Failure in Multiple Myeloma. N. Engl. J. Med..

[13] Garnett E.S., Goddard B.A., Markby D., Webber C.E. (1969). The spleen as an arteriovenous shunt. Lancet.

[14] Mendes F.R., Sobral K.M., Culler H.F., Campanelli Freitas Couto S., Pereira J., Rocha V., Martinez G.A., de Padua Covas Lage L.A. (2020). Acquired hemophagocytic lymphohistiocytosis as initial manifestation of multiple myeloma. A case report and literature review. Medicine.

[15] Gau Y.-C., Hsiao H.-H., Liu Y.-C., Yeh T.-J. (2020). Case report of coexistence of myeloproliferative neoplasms and multiple myeloma. Kaohsiung J. Med. Sci..

[16] Buzalewski J., Fisher M., Rambaran R., Lopez R. (2019). Splenic rupture secondary to amyloid light-chain (AL) amyloidosis associated with multiple myeloma. J. Surg. Case Rep..

[17] Tamir R., Lewin R.F., Inbal A., Heller I., Theodor E. (1985). High-output cardiac failure as a presenting symptom of plasma cell leukemia. Isr. J. Med. Sci..

[18] Chaoui D., Gallet B., Genet P., Mbungani B., Al Jijakli A., Arakelyan N., Mesbah L., Sutton L. (2013). High-output cardiac failure revealing primary plasma cell leukemia. Case Rep. Hematol..

[19] Giuliani N., Colla S., Rizzoli V. (2004). Angiogenic switch in multiple myeloma. Hematology.

[20] Ribatti D., Nico B., Vacca A. (2006). Importance of the bone marrow microenvironment in inducing the angiogenic response in multiple myeloma. Oncogene.

[21] Sasaki K., Yamashita K., Miyoshi T., Furukawa Y., Kimura T., Kita T., Ichinohe T., Ishikawa T., Sasada M., Uchiyama T. (2007). Analysis of serum angiogenic factors in young multiple myeloma patient with high-output cardiac failure. Int. J. Hematol..

[22] Ria R., Reale A., De Luisi A., Ferrucci A., Moschetta M., Vacca A. (2011). Bone marrow angiogenesis and progression in multiple myeloma. Am. J. Blood Res..

[23] Giuliani N., Storti P., Bolzoni M., Dalla Palma B., Bonomini S. (2011). Angiogenesis and multiple myeloma. Cancer Microenviron..

[24] De Luisi A., Binetti L., Ria R., Ruggieri S., Berardi S., Catacchio I., Racanelli V., Pavone V., Rossini B., Vacca A. (2013). Erythropoietin is involved in the angiogenic potential of bone marrow macrophages in multiple myeloma. Angiogenesis.

[25] Vacca A., Ria R., Reale A., Ribatti D. (2014). Angiogenesis in multiple myeloma. Chem. Immunol. Allergy.

[26] Ribatti D., Vacca A. (2016). Role of endothelial cells and fibroblasts in multiple myeloma angiogenic switch. Cancer Treat. Res..

[27] Ribatti D., Vacca A. (2018). New insights in anti-angiogenesis in multiple myeloma. Int. J. Mol. Sci..

[28] Ribatti D., Nico B., Crivellato E., Roccaro A.M., Vacca A. (2007). The history of the angiogenic switch concept. Leukemia.

[29] Lee N., Lee H., Moon S.Y., Sohn J.Y., Hwang S.M., Yoon O.J., Youn H.S., Eom H.S., Kong S.Y. (2015). Adverse prognostic impact of bone marrow microvessel density in multiple myeloma. Ann. Lab. Med..

[30] Ribatti D., Tamma R., Vacca A. (2019). Mast cells and angiogenesis in human plasma cell malignancies. Int. J. Mol. Sci..

[31] Vacca A., Ribatti D. (2005). Bone marrow angiogenesis in multiple myeloma. Leukemia.

[32] Rajkumar S.V., Mesa R.A., Fonseca R., Schroeder G., Plevak M.F., Dispenzieri A., Lacy M.Q., Lust J.A., Witzig T.E., Gertz M.A. (2002). Bone marrow angiogenesis in 400 patients with monoclonal gammopathy of undetermined significance, multiple myeloma, and primary amyloidosis. Clin. Cancer Res..

[33] Akob C., Sterz J., Zavrski I., Heider U., Kleeberg L., Fleissner C., Kaiser M., Sezer O. (2006). Angiogenesis in multiple myeloma. Eur. J. Cancer.

[34] Kumar S., Witzig T.E., Timm M., Haug J., Wellik L., Kimlinger T.K., Greipp P.R., Rajkumar S.V. (2004). Bone marrow angiogenic ability and expression of angiogenic cytokines in myeloma: Evidence favoring loss of marrow angiogenesis inhibitory activity with disease progression. Blood.

[35] Ribatti D., Vacca A. (2009). The role of monocytes-macrophages in vasculogenesis in multiple myeloma. Leukemia.

[36] Otjacques E., Binsfeld M., Noel A., Beguin Y., Cataldo D., Caers J. (2011). Biological aspects of angiogenesis in multiple myeloma. Int. J. Hematol..

[37] Borsi E., Perrone G., Terragna C., Martello M., Dico A.F., Solaini G., Baracca A., Sgarbi G., Pasquinelli G., Valente S. (2014). Hypoxia inducible factor-1 alpha as a therapeutic target in multiple myeloma. Oncotarget.

[38] Borsi E., Terragna C., Brioli A., Tacchetti P., Martello M., Cavo M. (2015). Therapeutic targeting of hypoxia and hypoxia-inducible factor 1 alpha in multiple myeloma. Transl. Res..

[39] Bhaskar A., Tiwary B.N. (2016). Hypoxia inducible factor-1 alpha and multiple myeloma. Int. J. Adv. Res..

[40] Testa U., Saulle E., Castelli G., Pelosi E. (2016). Endothelial progenitor cells in hematological malignancies. Stem Cell Investig..

[41] Mitsiades N., Mitsiades C.S., Poulaki V., Chauhan D., Richardson P.G., Hideshima T., Munshi N.C., Treon S.P., Anderson K.C. (2002). Apoptotic signaling induced by immunomodulatory thalidomide analogs in human multiple myeloma cells: Therapeutic implications. Blood.

[42] Lu L., Payvandi F., Wu L., Zhang L.-H., Hariri R.J., Man H.-W., Chen R.S., Muller G.W., Hughes C.C.W., Stirling D.I. (2009). The anti-cancer drug lenalidomide inhibits angiogenesis and metastasis via multiple inhibitory effects on endothelial cell function in normoxic and hypoxic conditions. Microvasc. Res..

[43] Chang D.H., Liu N., Klimek V., Hassoun H., Mazumder A., Nimer S.D., Jagannath S., Dhodapkar M.V. (2006). Enhancement of ligand-dependent activation of human natural killer T cells by lenalidomide: Therapeutic implications. Blood.

[44] Bloedjes T.A., de Wilde G., Guikema J.E.J. (2021). Metabolic effects of recurrent genetic aberrations in multiple myeloma. Cancers.

[45] Roberts R.S., Hsu H.W., Lin K.D., Yang T.J. (1976). Amino acid metabolism of myeloma in culture. J. Cell Sci..

[46] Mercille S., Massie B. (1994). Induction of apoptosis in nutrient-deprived cultures of hybridoma and myeloma cells. Biotechnol. Bioeng..

[47] Puchades-Carrasco L., Lecumberri R., Martínez-López J., Lahuerta J.-J., Mateos M.-V., Prosper F., San-Miguel J.F., Pineda-Lucerna A. (2013). Multiple myeloma patients have a specific serum metabolomic profile that changes after achieving complete remission. Clin. Cancer Res..

[48] Wise D.R., DeBerardinis R.J., Mancuso A., Sayed N., Zhang X.-Y., Pfeiffer H.K., Nissim I., Daikhin E., Yudkoff M., McMahon S.B. (2008). Myc regulates a transcriptional program that stimulates mitochondrial glutaminolysis and leads to glutamine addiction. Proc. Natl. Acad. Sci. USA.

[49] Gao P., Tchernyshyov I., Chang T.C., Lee Y.-S., Kita K., Ochi T., Zeller K.I., De Marzo A.M., Van Eyk J.E., Mendell J.T. (2009). C-Myc suppression of MiR-23a/b enhances mitochondrial glutaminase expression and glutamine metabolism. Nature.

[50] Lin C.Y., Lovén J., Rahl P.B., Paranal R.M., Burge C.B., Bradner J.E., Lee T.I., Young R.A. (2012). Transcriptional amplification in tumor cells with elevated C-Myc. Cell.

[51] Matsuzaki H., Matsuno F., Yoshida M., Hata H., Okazaki K., Takatsuki K. (1992). Human myeloma cell line (KHM-4) established from a patient with multiple myeloma associated with hyperammonemia. Intern. Med..

[52] Caminal L., Castellanos E., Mateos V., Astudillo A., Moreno C., Diéguez M.A. (1993). Hyperammonaemic encephalopathy as the presenting feature of IgD multiple myeloma. J. Intern. Med..

[53] Matsuzaki H., Hata H., Sonoki T., Matsuno F., Kuribayashi N., Yoshida M., Nagasaki A., Murata H., Fujiyama S., Takatsuki K. (1995). Serum amino acid disturbance in multiple myeloma with hyperammonemia. Int. J. Hematol..

[54] Takimoto Y., Imanaka F., Hayashi Y., Morioka S. (1996). A patient with ammonia-producing multiple myeloma showing hyperammonemic encephalopathy. Leukemia.

[55] Martinelli G., Peccatori F., Ullrich B., Ghielmini M., Roggero E., Goldhirsch A. (1997). Clinical manifestation of severe hyperammonemia in patients with multiple myeloma. Ann. Oncol..

[56] Kuribayashi N., Matsuzaki H., Hata H., Yoshida M., Sonoki T., Nagasaki A., Kimura T., Okamoto K., Kurose M., Tsuda H. (1998). Multiple myeloma associated with serum amino acid disturbance and high output cardiac failure. Am. J. Hematol..

[57] Keller D.R., Keller K. (1998). Hyperammonemic encephalopathy in multiple myeloma. Am. J. Hematol..

[58] Pérez Retortillo J.A., Marco F., Amutio E., Conde E., Iriondo A., Zubizarreta A. (1998). Hyperammonemic encephalopathy in multiple myeloma. Haematologica.

[59] Otsuki T., Yamada O., Sakaguchi H., Ichiki T., Kouguchi K., Wada H., Hata H., Yawata Y., Ueki A. (1998). In vitro excess ammonia production in human myeloma cell lines. Leukemia.

[60] Kwan L., Wang C., Levitt L. (2002). Hyperammonemic encephalopathy in multiple myeloma. N. Engl. J. Med..

[61] Holahan J.R. (2004). Hyperammonemia: Elevated ammonia levels in multiple myeloma. Am. J. Med..

[62] Shah A.S., Shetty N., Jaiswal S., Mehta B.C. (2005). Hyperammonemia: An unusual presenting feature of multiple myeloma. Indian J. Med. Sci..

[63] Furer V., Heyd J. (2007). Hyperammonemic encephalopathy in multiple myeloma. Isr. Med. Assoc. J..

[64] Talamo G., Cavallo F., Zangari M., Barlogie B., Lee C.-K., Pineda-Roman M., Kiwan E., Krishna S., Tricot G. (2007). Hyperammonemia and encephalopathy in patients with multiple myeloma. Am. J. Hematol..

[65] Lora-Tamayo J., Palom X., Sarrá J., Gasch O., Isern V., Fernández de Sevilla A., Pujol R. (2008). Multiple myeloma and hyperammonemic encephalopathy: Review of 27 cases. Clin. Lymphoma Myeloma.

[66] Ikewaki J., Ogata M., Imamura T., Kohno K., Nakayama T., Kadota J.-I. (2009). Development of hyperammonemic encephalopathy in patients with multiple myeloma may be associated with the appearance of peripheral blood myeloma cells. Leuk. Lymphoma.

[67] Bénet B., Alexandra J.-F., Andriu V., Sedel F., Ajzenberg N., Papo T. (2010). Multiple myeloma presenting as hyperammonemic encephalopathy. J. Am. Geriatr. Soc..

[68] Pham A., Reagan J.L., Castillo J.J. (2013). Multiple myeloma-induced hyperammonemic encephalopathy: An entity associated with high in-patients mortality. Leuk. Res..

[69] Li Y., Zhou Q., Song J.-N., Chen X.-Z., Zhang X.-Z., Sun Y. (2021). Analysis of clinical prognosis in patients with non-hepatic hyperammonemia. Medicine.

[70] Murray R.K., Granner D.K., Mayes P.A., Rodwell V.W. (2000). Harper’s Biochemistry.

[71] Krebs H.A., Veech R.L. (1970). Regulation of the redox state of pyridine nucleotids in rat liver. Pyridine Nucleotide Dependent Dehydrogenases.

[72] Meister A., Boyer P.D. (1974). Glutamine Synthetase of Mammals. The Enzymes.

[73] Mayer B., Hemmens B. (1998). Biosynthesis and action of nitric oxide in mammalian cells. Trends Biochem. Sci..

[74] Kovamees O., Shemykin A., Eriksson M., Angelin B., Pernow J. (2016). Arginase inhibition improves endothelial function in patients with familial hypercholesterolaemia irrespective of their cholesterol levels. J. Intern. Med..

[75] Mahdi A., Kovamees O., Checa A., Wheelock C.E., von Heijne M., Alvarsson M., Pernow J. (2018). Arginase inhibition improves endothelial function in patients with type 2 diabetes mellitus despite intensive glucose-lowering therapy. J. Intern. Med..

[76] Kwaan H.C. (2013). Hyperviscosity in plasma cell dyscrasias. Clin. Hemorheol. Microcirc..

[77] Uggla B., Nilsson T.K. (2015). Whole blood viscosity in plasma cell dyscrasia. Clin. Biochem..

[78] Dumas G., Merceron S., Zafrani L., Canet E., Lemiale V., Kouatchet A., Azoulay E. (2015). Hyperviscosity syndrome. Rev. Med. Interne..

[79] Caimi G., Carlisi M., Montana M., Gallà E., Lo Presti R., Hopps E., Siragusa S. (2018). Erythrocyte deformability and hemorheological profile in multiple myeloma. Clin. Hemorheol. Microcirc..

[80] Caimi G., Hopps E., Carlisi M., Montana M., Gallà E., Lo Presti R., Siragusa S. (2018). Hemorheological parameters in Monoclonal Gammopathy of Undetermined Significance (MGUS). Clin. Hemorheol. Microcirc..

[81] Caimi G., Lo Presti R., Carlisi M. (2021). Reflections on the unexpected laboratory finding of hemorheological alterations observed in some hematological disorders. Microvasc. Res..

[82] Carlisi M., Mancuso S., Lo Presti R., Siragusa S., Caimi G. (2021). Comparison between whole blood viscosity measured and calculated in subjects with monoclonal gammopathy of undetermined significance and in patients with multiple myeloma: Re-evaluation of our survey. Clin. Hemorheol. Microcirc..

[83] Popel A.S., Johnson P.C. (2005). Microcirculation and hemorheology. Annu. Rev. Fluid Mech..

[84] Gori T., Forconi S., Baskurt O.K., Hardeman M.R., Rampling M.W., Meiselman H.J. (2007). Endothelium and hemorheology. Handbook of Hemorheology and Hemodynamics.

[85] Forconi S., Wild P., Munzel T., Gori T. (2011). Endothelium and hyperviscosity. Clin. Hemorheol. Microcirc..

[86] Forconi S., Gori T. (2013). Endothelium and hemorheology. Clin. Hemorheol. Microcirc..

[87] Tsai A.G., Acero C., Nance P.R., Cabrales P., Frangos J.A., Buerk D.G., Intaglietta M. (2005). Elevated plasma viscosity in extreme hemodilution increases perivascular nitric oxide concentration and microvascular perfusion. Am. J. Physiol. Heart Circ. Physiol..

[88] Cabrales P., Martini J., Intaglietta M., Tsai A.G. (2006). Blood viscosity maintains microvascular conditions during normovolemic anemia independent of blood oxygen-carrying capacity. Am. J. Physiol. Heart Circ. Physiol..

[89] Katritsis D., Kaiktsis L., Chaniotis A., Pantos J., Efstathopoulos E., Marmarelis V. (2007). Wall shear stress: Theoretical considerations and methods of measurement. Prog. Cardiovasc. Dis..

[90] Sriram K., Intaglietta M., Tartakovsky D.M. (2014). Non-Newtonian flow of blood in arterioles: Consequences of wall shear stress measurements. Microcirculation.

[91] Zhou J., Li Y.-S., Chien S. (2014). Shear stress-initiated signaling and its regulation of endothelial function. Arterioscler. Thromb. Vasc. Biol..

[92] Uzarski J.S., Scott E.W., McFetridge P.S. (2013). Adaptation of endothelial cells to physiologically-modeled, variable shear stress. PLoS ONE.

[93] Abe J.-I., Berk B.C. (2014). Novel mechanisms of endothelial mechanotransduction. Arterioscler. Thromb. Vasc. Biol..

[94] Balligand J.L., Feron O., Dessy C. (2009). eNOS activation by physical forces: From short-term regulation of contraction to chronic remodeling of cardiovascular tissues. Physiol. Rev..

[95] Sriram K., Laughin J.G., Rangamani P., Tartakovsky D.M. (2016). Shear-induced nitric oxide production by endothelial cells. Biophys. J..

